# Combining Radiotherapy With Anti-angiogenic Therapy and Immunotherapy; A Therapeutic Triad for Cancer?

**DOI:** 10.3389/fimmu.2018.03107

**Published:** 2019-01-14

**Authors:** Ruben S. A. Goedegebuure, Leonie K. de Klerk, Adam J. Bass, Sarah Derks, Victor L. J. L. Thijssen

**Affiliations:** ^1^Amsterdam UMC, Location VUmc, Medical Oncology, Cancer Center Amsterdam, Amsterdam, Netherlands; ^2^Department of Medical Oncology, Dana-Farber Cancer Institute, Boston, MA, United States; ^3^Cancer Program, The Broad Institute of MIT and Harvard, Cambridge, MA, United States; ^4^Amsterdam UMC, Location VUmc, Radiation Oncology, Cancer Center Amsterdam, Amsterdam, Netherlands

**Keywords:** radiation, immune response, angiogenesis, therapy, combination treatment, clinical trials, tumor microenvironment, cancer

## Abstract

Radiotherapy has been used for the treatment of cancer for over a century. Throughout this period, the therapeutic benefit of radiotherapy has continuously progressed due to technical developments and increased insight in the biological mechanisms underlying the cellular responses to irradiation. In order to further improve radiotherapy efficacy, there is a mounting interest in combining radiotherapy with other forms of therapy such as anti-angiogenic therapy or immunotherapy. These strategies provide different opportunities and challenges, especially with regard to dose scheduling and timing. Addressing these issues requires insight in the interaction between the different treatment modalities. In the current review, we describe the basic principles of the effects of radiotherapy on tumor vascularization and tumor immunity and vice versa. We discuss the main strategies to combine these treatment modalities and the hurdles that have to be overcome in order to maximize therapeutic effectivity. Finally, we evaluate the outstanding questions and present future prospects of a therapeutic triad for cancer.

## Introduction

Radiotherapy has been an integral part of cancer treatment for over a century. More than half of all cancer patients undergo radiotherapy at some stage during treatment, either with curative intent, or in a palliative setting once the possibility for cure has been lost (1, 2). Radiotherapy was introduced shortly after the discovery of X-rays and gamma-rays in the late nineteenth century. Patients with different types of cancer were treated with radiotherapy, resulting in a paradigm shift in cancer therapy (3, 4). Since then, the clinical benefit of radiotherapy continuously improved, both by technical advancements and by increased insight in the biology behind the radiation response. For example, optimized treatment planning and more precise delivery techniques have made it possible to safely increase the tumor-targeted radiation dose while sparing the surrounding normal tissues. In addition, research into the cellular effects of ionizing radiation has provided detailed understanding of e.g., the cell cycle, apoptosis and DNA repair. This has offered insight in optimal dose-scheduling of radiotherapy (3). For example, the advantages of delivering a high dose of irradiation in multiple smaller fractions was already recognized in the 1930's (5). Further research has resulted in the definition of “the five Rs of radiobiology” which represent five different cellular aspects that affect the efficacy of fractionated irradiation and that later have been exploited to develop combination therapies (6, 7) (Box 1).

Box 1 **The 5 Rs of radiotherapy.**

The 5 Rs of radiotherapy represent a conceptual framework that form the rationale behind fractionation of radiotherapy. The 5 Rs are: Repair, Redistribution, Reoxygenation, Repopulation, and Radiosensitivity. **Repair** is the one of the primary reasons to fractionate radiotherapy. By applying fractionated radiotherapy, normal cells have the opportunity to repair sublethal DNA damage between each fraction while cancer cells are unable to sufficiently repair DNA damage due to defective or suppressed repair pathways. **Redistribution** relates to the ability of cells to progress in the cell cycle. Cells in S-phase are typically radioresistant, while cells in late G_2_ and M phase are relatively sensitive. Fractionated application of irradiation increases the chance that cells that were in a radioresistant phase at one fraction have 'redistributed' to a radiosensitive phase at the following fraction. **Reoxygenation** is related to the dynamic and changing hypoxic status of tumor tissue. Fractionated radiotherapy increases the chance that all areas of the tumor tissue receive a dose of irradiation when oxygenation is improved. **Repopulation** refers to the increase in cell division that is seen in normal and cancer cells after radiation. Cells that proliferate between fractions increases the number of cells that have to be killed by radiotherapy. Consequently, repopulation is affected by the time between fractions. **Radiosensitivity** refers to the intrinsic radiosensitivity or radioresistance of different cell types. It influences the total dose that is required for a given level of damage.

Initially, radiobiology research was mainly focused on the cancer cells without appreciating the role of the tumor microenvironment. However, over the past decades it has become clear that components within the tumor microenvironment such as the tumor vascular bed and tumor infiltrating immune cells have a pivotal impact on radiotherapy efficacy (5). For instance, radiotherapy can exert opposing effects on tumor vascularization and perfusion depending on dose-scheduling (8, 9). In addition, the abscopal effect, i.e. the observation that local tumor irradiation can also lead to regression of distant tumor masses, has been linked to the immune system (10). Consequently, both anti-angiogenic therapy and immunotherapy are evaluated in combination with radiotherapy. In the current review, we describe the basic concepts of the interactions between radiotherapy and the tumor vasculature as well as between radiotherapy and the tumor immune microenvironment. In addition, we discuss how both anti-angiogenic therapy and immunotherapy can influence the efficacy of radiotherapy and how a therapeutic triad might emerge as a powerful anti-cancer treatment modality.

## Radiotherapy and the Tumor Vasculature

The relation between radiotherapy and tumor vascularization has become apparent when it became clear that the effects of ionizing radiation largely depend on the generation of reactive oxygen species (ROS) (11). These highly reactive oxygen radicals can induce irreparable DNA damage that eventually leads to cancer cell death. As the generation of ROS depends on oxygen availability, well-vascularized and perfused tumor tissues are more susceptible to ionizing radiation. Thus, radiation damage is positively correlated with oxygen availability and while lack of oxygen, e.g., in hypoxic tumors, hampers treatment efficiency (11, 12). Indeed, a clinical study in patients with head and neck squamous cell carcinoma (HNSCC) comparing tumors with a median oxygen tension below and above 10 mmHg, reported disease free survival rates after radiotherapy of 22 vs. 78%, respectively (13). Furthermore, the uptake of hypoxia PET tracers has been reported to be of prognostic value for response evaluation (14). In line with this, it has been shown that tumor perfusion is a predictive factor for radiotherapy efficacy. Measuring blood flow and blood volume using either perfusion CT or the apparent diffusion coefficient with diffusion weighted MRI, has been found to predict the response to radiotherapy in patients with HNSCC (15, 16). Similar results were reported in patients with rectal cancer or cervical cancer (17, 18). These findings indicate that monitoring tumor perfusion and/or oxygenation prior to radiotherapy can be of value for setting up a proper treatment plan. This requires robust and reproducible imaging protocols as well as validated imaging biomarkers (14, 19). Modern PET/CT radiotherapy simulators already offer FDG-PET and dynamic contrast-enhanced CT imaging for a combined volumetric assessment of tumor metabolism and perfusion (14). With the current advances of MRI-guided adaptive radiotherapy, real time evaluation of tumor perfusion for predicting and monitoring treatment response might also become available. To what extent the clinical implementation of such techniques is feasible awaits further studies.

Apart from predicting treatment outcome, measuring tumor perfusion and oxygenation might also be of value to monitor the response during radiotherapy. Especially since perfusion not only affects radiotherapy, but radiotherapy also affects perfusion. The latter is related to the effects of radiotherapy on the vasculature, which are complex and appear to be dependent on the dose and scheduling of radiotherapy. Based on a literature review, Park et al. concluded that high dose irradiation, i.e., a dose above 10 Gy, induces acute vascular damage leading to deterioration of the tumor microenvironment and indirect cancer cell death (9). This was recently confirmed in a study showing that irradiation with a dose of 15–30 Gy resulted in dose-dependent secondary cell death. This was not observed after low-dose radiotherapy and most likely caused by vascular damage (20). Possibly, the vascular damage was caused by endothelial cell apoptosis, which can be induced by the upregulation of acid sphingomyelinase production in endothelial cells after high dose irradiation (21, 22).

Interestingly, fractionated low dose radiotherapy, i.e., daily fractions of up to 2 Gy, appears to exert a positive effect on the tumor vasculature and tissue perfusion (9, 23, 24) in multiple tumor models (25–27) as well as in patients (28–33). For example, an increased tumor blood volume during treatment with chemoradiation (27 × 1.8 Gy) was observed in cervical cancer patients (34). Using dynamic contrast-enhanced MRI and contrast-enhanced ultrasonography, we recently also observed increased tumor perfusion following two weeks of fractionated irradiation in a xenograft mouse tumor model. This was accompanied by reduced intratumoral hypoxia and increased tumor viability (35). Of note, increased tumor oxygenation during radiotherapy has been linked to different mechanisms, such as decreased oxygen consumption and vasorelaxation via increased inflammation (36). In addition, fractionated low dose irradiation can promote the growth of new blood vessels which might also contribute to enhanced perfusion, as discussed in the next section (23, 35, 37).

**Figure 1:**
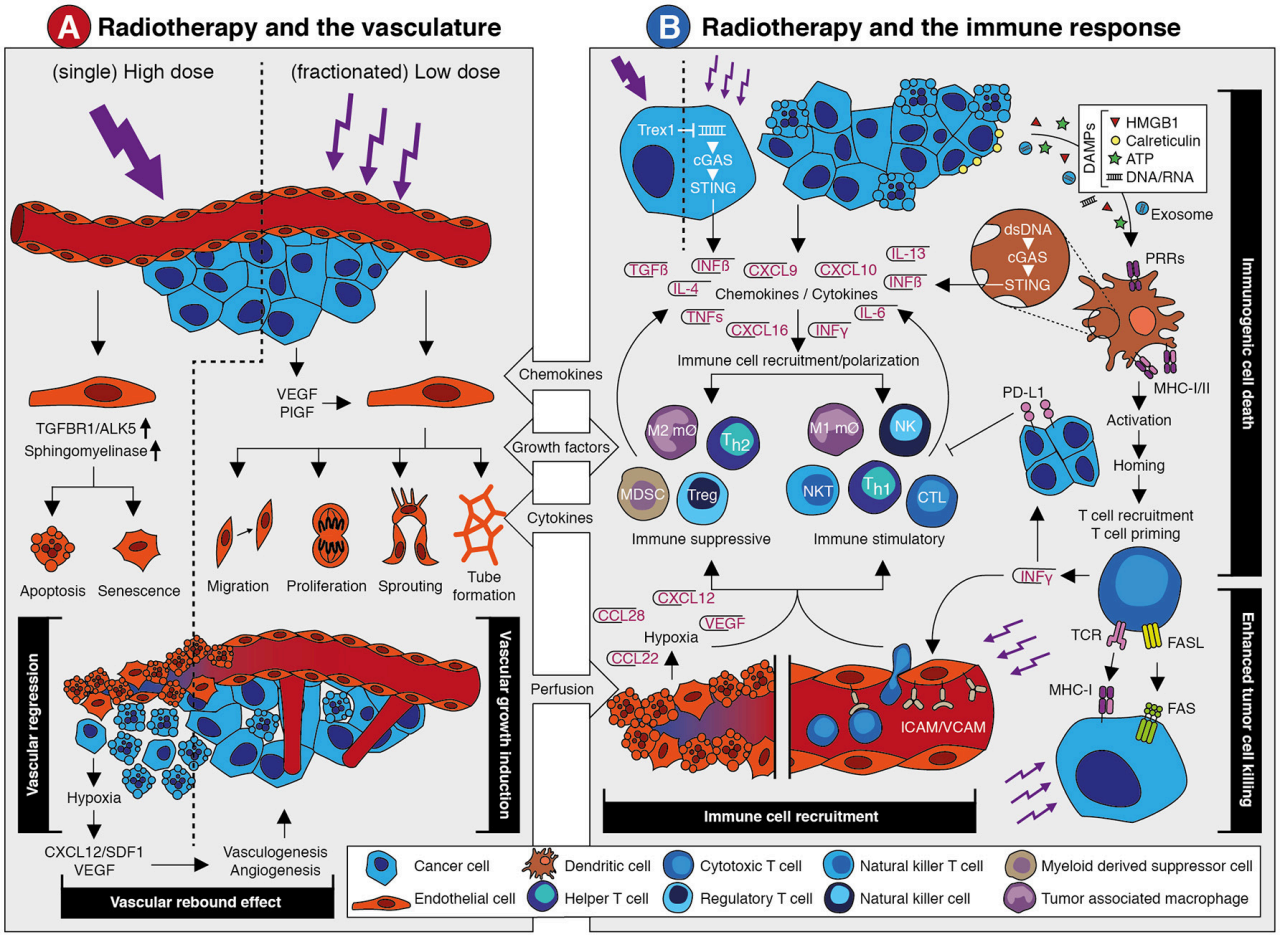
The effects of radiotherapy on the vasculature and the immune response. **(A)** Schematic overview of the main effects that occur in the vasculature in response to radiotherapy. A detailed description is provided in the main text. In brief, single high dose irradiation induces endothelial cell apoptosis and senescence via increased ALK5 and Sphingomyelinase expression. This causes vessel regression and vascular collapse which is accompanied by reduced perfusion. This eventually results in tissue hypoxia which leads to a vascular rebound effect by growth factor-induced vasculogenesis and angiogenesis. Fractionated low dose irradiation also induces an increased expression of angiostimulatory growth factors like VEGF and bFGF. This promotes different endothelial cell functions that results in vascular growth induction and enhanced tissue perfusion. Both the vascular rebound effect and vascular growth induction provide opportunities for therapeutic intervention in combination with radiotherapy. **(B)** Schematic overview of the main effects that occur in the vasculature in response to radiotherapy. A detailed description is provided in the main text. In brief, irradiation of tumor cells can induce expression of interferon beta (IFNβ) through cytosolic dsDNA/cGAS/STING signaling. This is dependent on dosing, as high dose irradiation induces Trex1 which causes clearance of cytosolic dsDNA. Apart from IFNβ, radiotherapy induces the expression and release of several chemokines, cytokines and growth factors that promote the recruitment of immune cells. This includes both suppressive and stimulatory immune cell subsets. At the same time, irradiation promotes an immune response via the induction of immunogenic cell death. The release of damage-associated molecular patterns (DAMPs) upon radiotherapy-induced cell death causes the activation of antigen presenting cells like dendritic cells through pattern recognition receptors (PPR). This eventually results in the recruitment and priming of cytotoxic T cells. This is accompanied by the release of cytokines like interferon gamma (IFNγ) which exerts diverging effects on the immune response. At one hand, IFNγ induces PD-L1 expression on tumor cells which is immunosuppressive. At the other hand, it stimulates the expression of leukocyte adhesion molecules in the vessel wall which contributes to increased immune cell recruitment. Vessel regression induces hypoxia which increases expression of growth factors and chemokines that affect immune cell recruitment and polarization. Finally, radiotherapy induces the expression of molecules on the tumor cell surface like MHC-I and Fas, which increases tumor cell killing by immune cells. Targeting the immune suppressive mechanisms provide opportunities for therapeutic intervention in combination with radiotherapy.

Collectively, there is clear evidence of a reciprocal relation between radiotherapy and the tumor vasculature in which an adequate tumor vascularization enhances radiotherapy efficacy, while irradiation induces dose-dependent effects on the vasculature (Summarized in Figure 1A). Exploiting this relation for combination therapies with angioregulatory strategies appears both feasible and challenging, especially with regard to dose scheduling.

## Combining Radiotherapy and Vascular Targeted Therapy

As described previously, proper tumor oxygenation is an important predictor of radiotherapy efficacy. Therefore, modification of tumor hypoxia and perfusion in order to enhance the clinical benefit of radiotherapy has been explored using different strategies. A straightforward approach to counteract a hypoxic tumor environment involves the use of hyperbaric oxygen or of hypoxic sensitizers like nitroimidazoles. Both strategies can result in a treatment benefit, as shown in a meta-analysis with HNSCC patients (38). Unfortunately, data on other tumor types is scarce (11). Today, neither hyperbaric oxygen nor nitroimidazoles have been implemented in routine clinical practice due to the small benefit in relation to either practical difficulties or toxicity. Accelerated radiotherapy with carbogen and nicotinamide (ARCON) is a more recent development, in which radiotherapy is combined with inhalation of a hyperoxic gas and a vasoactive agent, thereby decreasing both perfusion-limited hypoxia as well as diffusion-limited hypoxia in the lungs (39). Although promising, results of clinical trials are not conclusive with respect to local tumor control (40, 41). Vasodilating agents, such as nitric oxide, calcium antagonists and hydralazine, have also been studied as an approach to improve tumor perfusion in order to enhance radiotherapy efficacy, as reviewed by Sonveaux (42). However, both variable effects on radiosensitivity as well as the mutual systemic effects preclude their clinical use. To date, the most effective method to improve tumor perfusion in a clinical setting appears to be hyperthermia. While hyperthermia can promote cell death via induction of apoptosis or mitotic catastrophy, it has also been shown to improve the efficacy of radiotherapy by inhibition of DNA damage repair pathways and enhancement of tissue perfusion and oxygenation (43–45).

A somewhat unexpected method that was discovered to improve tumor perfusion and oxygenation is anti-angiogenic therapy. Anti-angiogenic therapy refers to treatment strategies that aim to block or hamper angiogenesis, i.e., the growth of new blood vessels of pre-existing capillaries (Box 2). It was proposed as an effective anti-cancer therapy in the early 1970's by prof. J. Folkman after his discovery that the growth of most solid tumors is dependent on angiogenesis (47). Initially, it was anticipated that anti-angiogenic drugs would hamper the effect of radiotherapy due to decreased perfusion and oxygenation. However, multiple preclinical studies observed an enhanced effect of the combinatorial approach (48–50). These findings have been confirmed in multiple conducted clinical trials investigating the combinatorial approach. For example, in a phase I study in patients with locally advanced pancreatic cancer, the vascular endothelial growth factor (VEGF) blocker bevacizumab displayed acceptable toxicity in combination with radiotherapy and capecitabine. Interestingly, only one of the 46 patients had progressive disease and median survival from the start of the protocol was 11.6 months (51). Promising results were also reported when bevacizumab was combined with capecitabine, oxaliplatin and radiotherapy in patients with rectal cancer (52). Thus far, the results from larger and more recent clinical trials are less conclusive, reporting variable efficacy as well as increasing toxicity [extensively reviewed by us previously 53, 54].

Box 2 **Angiogenesis.**

Angiogenesis is the growth of new blood vessels out of pre-existing capillaries. It is one of the hallmarks of cancer since most solid tumor cannot grow beyond a few cubic millimeters if they are unable to induce angiogenesis. The key players in the angiogenic process are endothelial cells. These cells form the inner lining of all blood vessels. Under hypoxic conditions, cancer cells undergo the so-called “angiogenic switch” which results in an elevated expression and secretion of soluble factors like vascular endothelial growth factor (VEGF). Secreted VEGF binds to it receptors on surface of endothelial cells in a nearby capillary vessel. As a result, the endothelial cells become activated and secrete proteases that degrade the capillary basement membrane as well as the underlying extracellular matrix. Subsequently the activated endothelial cells to proliferate and migrate into the direction of the growth factor gradient, thereby forming novel vascular sprouts toward the tumor that will eventually reassemble into a capillary bed. Due to an imbalance between angiostimulatory and angioinhibitory factors, the newly formed vasculature is abnormally structured, dysfunctional and unable to adequately relief tumor hypoxia. As a consequence, the pro-angiogenic stimulus is maintained and endothelial cells lose some of their typical functional features, including the expression of adhesion molecules that regulate the extravasation of leukocyte into the tumor tissue [For an extensive review see (46)].

While the clinical observations warrant further investigation regarding therapy optimization, the potential positive interaction between radiotherapy and anti-angiogenic therapy has been attributed to several distinct mechanisms, such as vessel normalization and the vascular rebound effect. The concept of vessel normalization was coined by prof. R. Jain to explain the paradoxical observation that drugs aimed at vessel pruning could in fact enhance the effect of therapies that rely on a functional vasculature, including radiotherapy (55). Based on the premise that the tumor vasculature is abnormally structured and dysfunctional due to a continuous imbalance between pro- and anti-angiogenic signaling, it was suggested that anti-angiogenic therapy restores the angiogenic balance thereby improving vessel function and tissue perfusion (55). Normalization of the tumor vasculature would thus result in enhanced tumor oxygenation and thereby increase the efficacy of radiation therapy. Indeed, transient improvement of hypoxia and pericyte coverage was reported in different tumor models treated with either a VEGF-receptor 2 blocking antibody, or a VEGF-receptor tyrosine kinase inhibitor (56, 57). Dings et al. (58) also studied tumor oxygenation in multiple tumor models during treatment with different anti-angiogenic drugs. Treatment with either bevacizumab or the anti-angiogenic peptide anginex induced elevated oxygenation levels and increased pericyte coverage in the first 4 days (58). Moreover, the anti-tumor effect improved when radiotherapy was applied within the window of increased oxygenation (57, 58).

While the previous findings indicate that vascular normalization could improve tumor perfusion, it has also become clear that vascular normalization occurs only transiently and that continuation of anti-angiogenic treatment eventually causes vessel regression and reduced tumor oxygenation (57–60). This has important therapeutic consequences, especially since the data on the exact occurrence and timing of the vascular normalization window in patients is limited (61–63). Characteristic features of vessel normalization like reduction of immature vessels and increased pericyte coverage have been observed in patient treated with bevacizumab (64). Furthermore, improved perfusion has been reported in a subset of glioblastoma multiforme (GBM) patients treated with cediranib (a pan-VEGF TKI) or cediranib-containing regimens, and was associated with survival benefit (61, 65). Notwithstanding these latter observations, the temporary character of vessel normalization in mice, i.e., a few days, seems to be in contrast with the beneficial effects for patients receiving anti-angiogenic drugs during several weeks of fractionated irradiation. Moreover, anti-angiogenic therapy is not only beneficial when applied prior to radiotherapy but also when given during or after radiotherapy (54). Thus, although vessel normalization might partially explain the beneficial effects, other mechanisms might be equally relevant for the interaction between both radiotherapy and anti-angiogenic therapy.

Another possible mechanism that could explain the benefit of anti-angiogenic drugs involves the stimulation of angiogenesis by irradiation, referred to as the vascular rebound effect. As described previously, low dose irradiation has been found to increase tumor perfusion and oxygenation. While this was linked to mechanisms such as vasodilation by enhanced inflammation and reduced oxygen consumption (36), we and others have shown that low-dose irradiation can also influence angiogenesis by inducing the expression of pro-angiogenesis growth factors like VEGF by cancer cells or other cells that reside in the tumor microenvironment (35, 66–68). For example, Sofia-Vala et al. (23) showed that low dose irradiation induces VEGF signaling in endothelial cells. Likewise, macrophages in the stromal tissue have been shown to enhance their VEGF expression after irradiation (69). We observed induction of VEGF and PlGF after 2 weeks of fractionated irradiation (daily fractions of 2 Gy) in cultured cancer cells as well as in xenograft tumor tissues (37). The induction of VEGF coincided with increased tumor perfusion, increased tissue viability and reduced hypoxia. In addition, the levels of VEGF were sufficient to stimulate endothelial cell migration and sprouting. Importantly, the anti-angiogenic drug sunitinib, which blocks VEGF-dependent signaling, could hamper these effects (37). These findings suggest that ionizing radiation can enhance tumor perfusion by induction of a pro-angiogenic response which can be counteracted by anti-angiogenesis treatment (35). Interestingly, when exploring the optimal dose-scheduling of fractionated low-dose radiotherapy with sunitinib, a small molecule that inhibits multiple tyrosine kinase receptors including VEGFR, we observed that the beneficial effects of the combination treatment could be obtained with a lower dose of anti-angiogenic drugs than what is currently applied for cancer treatment (35, 54). A similar observation was made by Wachsberger et al. (70) using VEGFtrap, a soluble receptor that “traps” VEGF. These findings are clinically relevant since the implementation of combination therapy is currently restricted due to increased toxicity in tumor types such as rectal cancer, nasopharyngeal cancer and glioblastoma (53). Of note, high dose irradiation can also induce a vascular rebound effect due to the vascular collapse and subsequent tissue hypoxia. In addition, intermediate and high dose irradiation have been suggested to trigger vasculogenesis, i.e., the influx of endothelial progenitor cells from other parts of the body or bone marrow to build vessels (71). This process is mediated via various chemokines including CXCL12/SDF1. Interfering in this process by blocking the CXCL12/SDF1 receptor (CXCR4) could be of interest in relation to radiotherapy (72). Furthermore, recent research on the role of endothelial cell metabolism in cancer have led to new insights and potential targets for anti-angiogenesis therapy. For example, inhibition of PFKFB3, which is a regulator of glycolysis, can promote vessel normalization, albeit that this effect is dose-dependent (73). Whether and to what extend such inhibitors synergize with radiotherapy awaits further investigation.

Collectively, the findings described above point toward the importance of proper dose-scheduling of both treatment modalities to achieve optimal beneficial effects. On one side, the dose-scheduling of anti-angiogenic drugs influences whether and when vessel normalization occurs and whether and when the angiogenic rebound effect is countered. On the other side, the dose-scheduling of radiotherapy influences whether and when tumor perfusion is affected and whether and when an angiogenic (rebound) effect occurs. This complex relation illustrates the challenges that accompany the combination of radiotherapy with anti-angiogenic therapy. It also explains that, while a plethora of pre-clinical evidence suggests a treatment benefit for the combination of radiotherapy with anti-angiogenic therapy, the clinical practice is less conclusive. The radiotherapy efficacy might be strengthened by a pro-angiogenic response, enhancing both tumor perfusion and oxygenation but this could at the same time induce unwanted tumor growth. Thus, optimal dose-scheduling of both treatment modalities is key to achieve beneficial effects and limit toxicity of the combination therapy.

## Radiotherapy and the Immune System

The link between radiotherapy and the immune system was recognized already several decades before the role of the tumor vasculature was uncovered. The first clear observation that the host immune system contributes to radiotherapy efficacy was presented in the late seventies of the previous century. In a preclinical study it was shown that the effect of radiotherapy is compromised in immunodeficient and CD8+ T cell depleted mice (74). Prior to this, radiotherapy was more or less considered to be immunosuppressive (75, 76). Additional evidence for a role of the immune system during radiotherapy was obtained from preclinical research and multiple case studies that reported on regression of (metastatic) tumor masses that were distant from the irradiated site (77–79). This so-called abscopal effect (Box 3) was already described in 1953, but it took about 50 years to link this to a systemic anti-tumor immune response initiated by radiotherapy (80, 81). Still, the exact mechanisms behind the abscopal effect are not entirely elucidated. Nevertheless, the clear link between radiotherapy and the immune response, together with the breakthrough of immunotherapy in recent years, has renewed the interest in combining radiotherapy and immunotherapy. Similar as for anti-angiogenic therapy, preclinical and clinical studies using this combination therapy have made it clear that successful implementation of radiotherapy combined with immunotherapy relies on a proper understanding of the interaction between both treatment modalities. In recent years, several mechanisms have been proposed that explain how radiotherapy affects the tumor immune response (82, 83) (Illustrated in Figure 1B).

Box 3 **The abscopal effect.**

The concept and term “abscopal” was proposed in 1953 by dr. R.H. Mole to describe effects of irradiation that occur distant from the site of irradiation, but within the same organism (78). The term originates from the prefix *ab-* (away from) and Latin word *scopus* (mark or target). As such, it can be considered as a systemic response following a local trigger. Today, the abscopal effect has been reported in a wide variety of both solid and hematologic tumor types. While the mechanism is still not fully elucidated, it has been established the abscopal effect involves the immune system [For an extensive review see (80)].

A well-recognized mechanism by which radiotherapy can enhance the anti-tumor immune response is the induction of immunogenic cell death. Unlike normal cell death, immunogenic cell death makes cancer cells visible to the immune system by the release of damage-associated molecular patterns (DAMPs), such as calreticulin, HMGB1 and ATP, along with the presentation of neoantigens and tumor associated antigens (84–91). DAMPs bind to pattern recognition receptors (PRRs) such as Toll-like receptors (TLRs) on antigen presenting cells, including dendritic cells (DCs). This leads to DC activation which subsequently cross-present antigens and migrate to the tumor-draining lymph node (92, 93), where they prime naive T cells and B cells to initiate a systemic immune response (92–99). Recent studies have identified the STING pathway, activated upon recognition of double-stranded DNA (dsDNA) via cytosolic DNA sensors, as an important regulator of this immunogenic cell death response (100–105). Double-stranded DNA can be transferred via exosomes from irradiated cancer cells to DCs. Subsequently, STING-dependent activation of type-I interferons and upregulation of co-stimulatory molecules is triggered (106). Collectively, these findings show that radiotherapy can promote an anti-tumor immune response via immunogenic cell death-mediated activation of antigen presenting cells like DCs leading to increased priming of tumor antigen-specific T cells.

Apart from enhanced T cell priming through immunogenic cell death, radiotherapy can also promote the trafficking of immune cells into the tumor. In fact, multiple mechanisms contribute to this enhanced immune infiltration. Firstly, radiotherapy can improve tumor perfusion (as described above) which will increase the number of leukocytes passing through the tumor tissue. Secondly, irradiation induces the endothelial expression of leukocyte adhesion molecules like ICAM and VCAM (93, 107–109). Consequently, leukocyte extravasation from the circulation into the tumor tissue will be increased. Thirdly, radiotherapy has been shown to increase the expression of pro-inflammatory chemokines such as CXCL9, CXCL10, and CXCL16 by cancer cells. This will help to attract leukocyte populations like cytotoxic CD8+ T cells, Th1 cells, NK cells, and NKT cells (108, 110, 111). Finally, radiation can induce MHC-I expression on cancer cells, either by an accumulation of damaged proteins and their break-down products (89, 97, 112), or in response to a general increase of IFN gamma (IFNγ) within the tumor microenvironment (108). Preclinical studies have also shown that radiotherapy enhances the expression of the death receptor Fas (CD95) on cancer cells, making them more susceptible to Fas ligand mediated cell death (97, 113–116). Altogether, enhanced tumor perfusion, increased leukocyte chemoattraction and extravasation, as well as increased susceptibility to T cell-mediated cell death contribute to an improved immune response during radiotherapy.

Unfortunately, there are some ifs and buts to the immunostimulatory effect of radiotherapy. Similar as with the angioregulatory response, the immunoregulatory response to irradiation appears to be dose and schedule dependent. For example, the induction of MHC-I (97, 112) and immunogenic cell death (89) depend on the dose, and in preclinical models moderate to high doses of radiotherapy seem to have most effect (92, 117, 118). For instance, Filatenkov et al. showed in weakly immunogenic CT26 and MC38 colon tumors that only a single dose of 30 Gy increased intratumoral CD8+ T cells, whereas 10 × 3 Gy did not (118). On the other hand, radiotherapy doses of ≥12 Gy have been shown to attenuate radiotherapy-induced tumor immunogenicity through the induction of DNA exonuclease TREX1 (Three prime repair exonuclease 1), which degrades cytosolic dsDNA, thereby preventing cGAS/STING mediated induction of interferon beta (IFNβ) (119). With regard to the abscopal effect, only a few comparative studies are available, but a systematic review of 46 case reports revealed a broad range in cumulative dose at which the effect was observed (range 0.45–60.75 Gy; median 31 Gy) (77). With regard to scheduling there is also no clear answer yet. It has been reported that a single fraction is better than multiple fractions (93), that there is no difference between single or multiple fractions (92), or that multiple fractions are better (120, 121). From a tumor perfusion perspective there is evidence that fractionated low dose is preferred over single high dose as described previously. At the same time, the induction of leukocyte adhesion molecule expression appears to be dose-dependent (109, 122, 123). So, a major future challenge will be to unravel at what dose-scheduling regime an optimal immunostimulatory effect of radiotherapy will occur.

Most likely, the overall effect of radiotherapy on the immune response is not only dose-scheduling dependent but is also determined by tumor type and the tumor microenvironment. Regarding the latter, it has been shown that the efficacy of radiotherapy is influenced by the composition of the pretreatment tumor immune microenvironment (124). Thus, it would be of interest to explore to what extent the pre-treatment immunogenic profile in the tumor tissue can predict the response to radiotherapy. This is also relevant given the observation that radiotherapy can induce an immunosuppressive microenvironment. After all, apart from the induction of pro-inflammatory chemokines, as described above, radiotherapy can also induce chemokines and cytokines that attract immunosuppressive cell populations such as Tregs (97), myeloid derived suppressor cells (MDSCs) (125), M2 macrophages, and Th2-skewed CD4+ T cells (126) to the tumor immune microenvironment (127). Multiple *in vitro* studies demonstrated that unpolarized macrophages tend to acquire a M1 phenotype after irradiation with 2–5 Gy. Interestingly, Klug et al. (128) showed in an *in vivo* model reprogramming of TAMs to a M1 phenotype after irradiation with 2 Gy. Different dose-effects of radiotherapy on TAMs, as well as mechanisms involved, has been described in detail by Genard et al. (129). Blockade of the macrophage chemoattractant CSF-1 and repolarization of macrophages into a M1 tumor suppressive phenotype by blocking interleukin-4 (IL-4) and IL-13 significantly improved responses to radiotherapy in a mouse breast cancer model (126, 130). In addition, IFN gamma expression within the tumor immune microenvironment is an important driver of PD-L1 expression on tumor and immune cell which leads to impairment of T cell function (131–133). In fact, it were these kind of observations that led to the hypothesis that the combination of immunotherapy with radiotherapy might have clinical benefit.

## Enhancement of Immunotherapy Efficacy by Radiotherapy

One of the major breakthroughs in oncology in recent years has been the development of drugs that enhance the potency of the immune system. These drugs are predominantly inhibitors of so-called immune checkpoint proteins (Box 4) and they are able to re-activate T cells to attack cancer cells. Although we are only starting to understand the effect of such immune checkpoint inhibitors, it has become clear that these drugs are most effective when the T cells that they activate are already in the tumor microenvironment (134–136). However, many tumors lack a proper lymphocyte infiltration. As described above, radiotherapy can elicit an anti-tumor T cell response, which has spurred the interest to apply radiotherapy in order to augment the local and systemic effect of immunotherapy. Evidence that radiotherapy can reliably and consistently achieve this effect in cancer patients is currently not available but multiple retrospective studies have shown that radiotherapy can increase the response to immunotherapy. Several studies [for overview see 137] in predominantly melanoma and lung cancer patients have shown that radiotherapy given during the course of immunotherapy increases the median overall survival compared to no radiotherapy (138, 139). Also in lung cancer it has been shown that radiotherapy somewhere in the course of the disease prior to the first cycle of PD-1 inhibitor pembrolizumab significantly increased overall and progression free survival (139). In metastatic non-small cell lung cancer (NSCLC) preliminary results of an ongoing trial (NCT02492568) with pembrolizumab preceded by stereotactic body radiation therapy showed a doubling of the overall response rate (140). However, other studies in melanoma and various solid tumors evaluating the combination of radiotherapy with ipilimumab (98) or pembrolizumab (141) showed disappointing results. The same holds true for a large phase III trial testing radiotherapy followed by ipilimumab or placebo in castration-resistant prostate cancer patients (142).

Box 4 **Immune checkpoint proteins.**

Immune checkpoints programmed cell death protein 1 (PD-1) and cytotoxic T-lymphocyte associated protein 4 (CTLA-4) are negative regulators of T cell responses and act as a brake on the immune system. Although CTLA-4 and PD-1 have similar negative effects on T cells activity, the immune checkpoints operate on different stages of an immune response. CTLA-4 expression is confined to T cells and functions mostly during the priming phase of T cell activation in lymph nodes. The PD-1 checkpoint is predominantly at play during the effector phase within peripheral tissues, where it interacts with its ligand PD-L1 which is broadly expressed on both tumor and immune cells. Despite these differences, inhibitors of both PD-1/PD-L1 and CTLA-4 are able to (re-)activate T cells to attack cancer cells and have shown unprecedented durable responses in many cancer types.

Interestingly, there is also a variety of case reports describing major systemic antitumor effects of palliative radiotherapy in patients that had progressed on immunotherapy. For instance, Postow et al. (94) showed, in a case report of a metastatic melanoma patient that had progressed under ipilimumab, re-induction of an anti-tumor immune response after palliative radiotherapy. This response was accompanied by the expansion of existing, and appearance of new anti-tumor antibodies (94). Another retrospective analysis of 21 patients with advanced melanoma who received radiotherapy after progression on ipilimumab showed partial systemic response and stable disease in 43% and 10% of cases, respectively (143). A beneficial effect of radiotherapy following progression on checkpoint inhibition has also been reported for a patient with NSCLC (144) and HNSCC (145). Another study of patients with stage IV melanoma treated with ipilimumab followed by palliative radiotherapy within the first 5 days of treatment showed that around 50% of patients experienced clinical benefit (146). Nevertheless, most clinical success of combined radiotherapy with immunotherapy has been shown in the adjuvant use of PD-1 pathway inhibitors. The largest study among those is the PACIFIC study, a multicenter randomized controlled trial comparing the use of PD-L1 inhibitor durvalumab as consolidation therapy following definitive chemoradiation in stage III NSCLC which showed a median progression free survival of 16.8 months compared to 5.6 months with placebo and an acceptable toxicity profile, resulting in prompt FDA approval of the adjuvant use of durvalumab for stage III NSCLC patients (147). Importantly, the combination of radiotherapy and immunotherapy appears to be safe and well tolerated without severe toxicities (138, 146–150). Altogether, these studies suggest a bright future for combined radiotherapy and immunotherapy for certain patients. Of note, the high expectations might be somewhat hampered by clinical studies that explored the concurrent use of immunotherapy and radiotherapy to stimulate an anti-tumor immune response by both modalities at the same time. Although the results of such studies are still in early phase, a recent phase I trial in patients with metastatic or locally advanced bladder cancer was paused early due to intolerable in-field toxicities (151). Trials to test the safety and feasibility of neoadjuvant immunotherapy with radiotherapy in NSCLC, HNSCC, and gastroesophageal cancer (NCT03245177, NCT03383094, and NCT03044613, respectively) amongst others are currently ongoing. Apparently, and in line with the observations of anti-angiogenic therapy combined with radiotherapy, the timing, dosing and scheduling of both treatments is key in achieving optimal therapeutic effects.

## Alternative Combined Radiotherapy-Immunotherapy Approaches

While currently most (pre)clinical research is mainly focused on the combination of radiotherapy with immune checkpoint inhibitors, several alternative immunomodulatory approaches are also being explored. For example, the combination of radiotherapy with immunostimulatory factors such as interleukin-2 (IL-2) (152, 153), granulocyte-macrophage colony-stimulation-factor (GM-CSF) (154), and agonists of the T cell co-stimulatory receptor OX40 (155, 156) has yielded promising responses in early phase clinical trials. Also strategies to trigger an anti-tumor immune response by intratumoral injection of TLR9 agonists in combination with concurrent low-dose radiotherapy on the injection site has shown promising results and excellent safety and tolerability in different tumor types, including low-grade B cell lymphomas (157), cutaneous T cell lymphoma (158) and follicular lymphoma (159). A TLR3 agonist in combination with concurrent fractionated radiotherapy was recently tested in a single arm phase II trial in 30 patients with newly diagnosed glioblastoma multiforme and was found to be well tolerated (160). Others have performed studies in which radiotherapy was combined with intratumoral injections of autologous immature DCs after radiotherapy in hepatocellular carcinoma (161) and soft tissue sarcoma (162). This treatment was also well tolerated and based on the observed responses, future phase II and III studies were recommended. Finally, efforts have been made to combine radiotherapy with vaccination against carcinoembryonic antigen (CEA) combined with GM-CSF in colorectal cancer (163), or against prostate specific antigen (PSA) combined with GM-CSF and IL-2 in patients with prostate cancer (164, 165). Despite the clear rationale behind these trials, both studies showed limited effectivity (163–165). On the other hand, a phase I clinical trial in chemo-naïve esophageal squamous cell carcinoma did show vaccine-specific cellular and clinical responses (CT evaluation) after treatment with a peptide vaccine containing five tumor-associated peptides (TTK, URLC10, KOC1, VEGFR1, and VEGFR2) in combination with chemoradiation (60 Gy, cisplatin, 5-FU) (166). All these studies exemplify the current interest and feasibility to combine radiotherapy with immunostimulatory treatments. Still, many questions have to be answered and challenges have to be met, especially with regard to dosing, scheduling and timing of both treatments. Nevertheless, the outlook for radiotherapy in combination with immunotherapy appears promising.

## Future Perspectives – A Therapeutic Triad

Based on aforementioned interactions and synergy, a trimodal approach combining radiotherapy with anti-angiogenic therapy and immunotherapy is a promising therapeutic strategy. To our best knowledge, no clinical trials have been published combining all three treatment modalities. Radiotherapy with either anti-angiogenic therapy or immunotherapy appears feasible, but presents both researchers and clinicians with many challenges.

**Figure 2:**
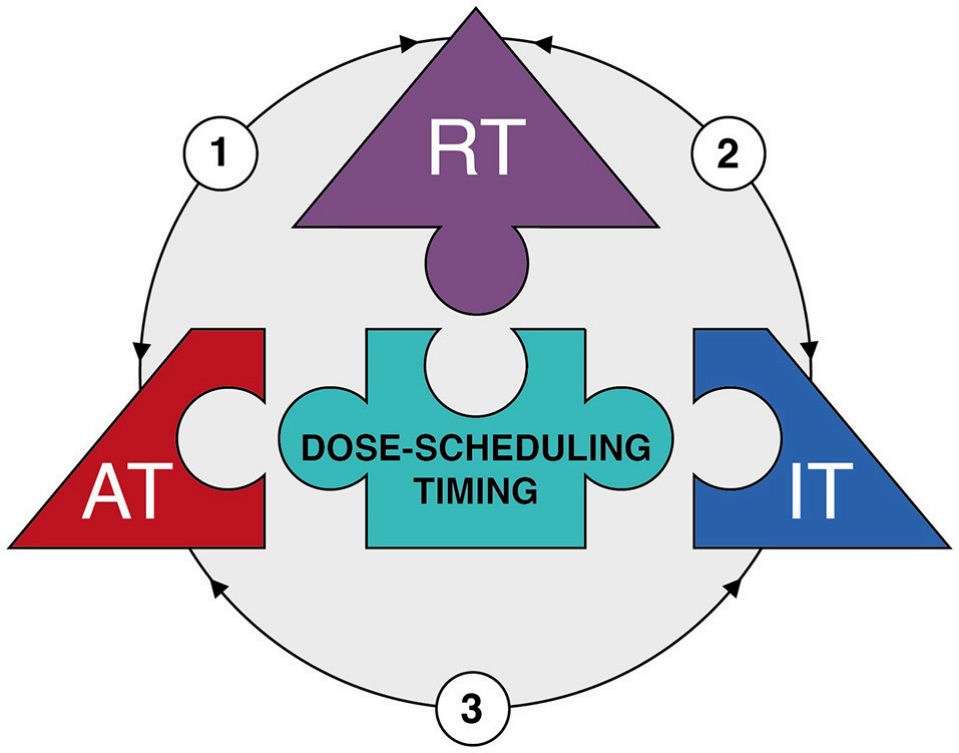
The therapeutic triad. Diagram depicting the main components of the 'therapeutic triad' as pieces of a jigsaw puzzle, i.e. radiotherapy (RT), anti-angiogenic therapy (AT), and immunotherapy (IT). Optimization of dose-scheduling and timing of the three treatment modalities is the center piece of the puzzle, for it is essential to achieve effective combination therapy with minimal toxicities. The arrows reflect the interactions between the different treatment modalities (see main text for more detailed information). In brief: (1) Radiotherapy has dose-dependent effects on tumor vessels resulting a vascular rebound effect due to either vascular collapse or direct induction of angiogenesis. This provides an opportunity for anti-angiogenic therapy. Anti-angiogenic therapy itself induces vessel normalization which improves tumor perfusion and oxygenation; this in turn enhances the efficacy of radiotherapy. (2) Radiotherapy induces immunogenic cell death which enhances specific T cell priming. In addition, radiotherapy can induce the expression of adhesion molecules on endothelial cell and chemokines by cancer cells which both improve the extravasation of immune cells into the tumor tissue. This enhances the efficacy of immunotherapy. In addition, the tumor immune microenvironment itself affects the response to radiotherapy. (3) Anti-angiogenic therapy induces vessel normalization which improves extravasation of immune cells into the tumor tissue. Likewise, immunotherapy might result in recruitment of immune subsets with angioregulatory activity which can be targeted by anti-angiogenic therapy.

While this review focused on the interaction of radiotherapy with either anti-angiogenic therapy or immunotherapy, there is growing awareness that the latter two treatments are also intrinsically interwoven. Indeed, the combination of immunotherapy and anti-angiogenic therapy has recently emerged as a novel therapeutic strategy (167). This is based on the observation that anti-angiogenic therapy can enhance immune effector cell trafficking to the tumor site. This would strengthen the efficacy of immunotherapy since low immune cell infiltration still represents a major obstacle for cancer immunotherapy (168). A recent review on this subject by Fukumura et al. (169) provides an up-do-date table of pre-clinical and clinical trials. The improved recruitment of immune cells during anti-angiogenic therapy is partly explained by vessel normalization. In the tumor endothelium, the expression of adhesion molecules that facilitate rolling, adhesion and extravasation of immune cells is reduced due to exposure of endothelial cells to tumor-derived angiogenic growth factors (170–172). This phenomenon is referred to as endothelial cell anergy and it makes the underlying tumor tissue invisible or at least less reachable to the immune system (173). In addition, hypoxia due to impaired perfusion results in the expression of several chemokines such as stromal cell–derived factor 1 (SDF1-α), CC-chemokine ligand 22 (CCL22) and CCL28. These chemokines initiate a state of tolerance by recruiting Tregs, MDSCs and M2-type TAMs to induce an immunosuppressive microenvironment (174, 175). Furthermore, hypoxia as well as VEGF can induce the expression of immune checkpoint molecules on cancer cells and immune cells (176, 177). Collectively, the hypoxic and pro-angiogenic tumor microenvironment are generally immunosuppressive. Thus, strategies that normalize the dysfunctional vasculature can not only restore immune cell functions and facilitate their antitumor activities, but also enhance immunotherapy effects (8). As already described, anti-angiogenic therapy can induce vascular normalization and reduce hypoxia. In line with this, anti-angiogenic drugs have been shown to facilitate tumor infiltration of CD8+ T lymphocytes and potentiate cancer immunotherapy (178–181). This effect could thus add up to the previously described induction of adhesion molecule expression in endothelial cells by radiotherapy itself. While anti-angiogenic therapy can influence the immune system, evidence is emerging that immunotherapy also affects the tumor vasculature. Interferon gamma is suggested to play an important role in this process, as it is produced by activated T cells and, upregulates ICAM-1 and induces T cell migration. Interestingly, Th1 cell infiltration is reported to reciprocally promote blood vessel normalization which would further contribute to an immunostimulatory microenvironment, in a process that is also dependent on IFNγ signaling. For example, in mice treated with anti PD-1 antibodies, Th1-mediated vessel normalization was improved (182). Thus, a mutual regulatory feedback loop is identified in which vessel normalization and T lymphocyte infiltration can amplify the positive effects conferred by each individual effect. Possibly, this combinatorial approach could lead to a more pronounced vessel normalization window which could be exploited to enhance the effect of radiotherapy. In this context it is noteworthy to mention that is has been shown in melanoma models that the improved immune response following STING activation actually depends on the production of IFNβ by endothelial cells (183). While this effect was observed after STING activation by intratumoral injection of cyclic dinucleotide GMP-AMP (cGAMP) and not by irradiation, it further indicates that targeting endothelial cells to improve immunotherapy could be of interest during radiotherapy. Thus, combining the three treatment modalities as a “therapeutic triad” offers an innovative and interesting approach to cancer treatment (Figure 2), but will even present with additional challenges regarding optimal dose-scheduling, timing and overcoming potential toxicities as compared to the combination of two treatments.

## Concluding Remarks

Although combining radiotherapy with either anti-angiogenic therapy or immunotherapy has been extensively studied the last decade, phase III studies showing a clear benefit of combinatorial approaches are scarce. This not only illustrates the complex relationship between the cancer cells and the tumor microenvironment, but it also emphasizes that many challenges have to be overcome to make these combination therapies effective. In particular, future studies should shed light upon the optimal timing and dosing of the different treatments. In addition, finding predictive and prognostic biomarkers could help determine which cancer types and disease stages are particularly suitable for combinatorial approaches. Interestingly, radiotherapy, anti-angiogenic therapy and immunotherapy all exert effects on both the tumor vasculature and the anti-tumor immune response. Better understanding of their reciprocal interactions in the tumor microenvironment is the main future challenge to allow the development of a therapeutic triad that combines the three treatment modalities for effective cancer therapy.

## Author Contributions

All authors listed have made a substantial, direct and intellectual contribution to the work, and approved it for publication.

### Conflict of Interest Statement

AB receives research funding from Merck and Novartis. The remaining authors declare that the research was conducted in the absence of any commercial or financial relationships that could be construed as a potential conflict of interest. The handling Editor declared a shared affiliation, though no other collaboration, with one of the authors LdK.
